# Designing community-based strategies to reach non-household contacts of people with tuberculosis in Lusaka, Zambia: a rapid qualitative study among key stakeholders

**DOI:** 10.3389/fpubh.2024.1408213

**Published:** 2025-01-13

**Authors:** Andrew D. Kerkhoff, Marksman Foloko, Evelyn Kundu-Ng’andu, Herbert Nyirenda, Zainab Jabbie, Mainza Syulikwa, Chanda Mwamba, Mary Kagujje, Monde Muyoyeta, Anjali Sharma

**Affiliations:** ^1^Division of HIV, Infectious Diseases and Global Medicine, Zuckerberg San Francisco General Hospital and Trauma Center, University of California San Francisco, San Francisco, CA, United States; ^2^Centre for Infectious Disease Research in Zambia, Lusaka, Zambia

**Keywords:** tuberculosis, active case finding, non-household contacts, community-based, stakeholder-engaged

## Abstract

**Background:**

In high-burden settings, most tuberculosis (TB) transmission likely occurs outside the home. Our qualitative study in Zambia explored the acceptability and preferences for designing TB active case finding (ACF) strategies to reach non-household contacts of people with TB.

**Methods:**

We conducted 56 in-depth interviews with persons with TB (*n* = 12), TB healthcare workers (HCWs) (*n* = 10), TB lay HCWs (*n* = 10), and leaders/owners (*n* = 12) and attendees (*n* = 12) of community venue types identified as potential TB transmission locations. Interviews explored TB attitudes and beliefs, and perceptions toward two ACF strategies targeting non-household contacts: (1) “social-network strategy”—persons with newly diagnosed TB reach out to their close non-household contacts and (2) “venue-based strategy”—HCWs conduct screening at community venues frequented by persons with newly diagnosed TB. We used the Consolidated Framework for Implementation Research (CFIR) framework to develop interview topic guides and analyze data using a rapid deductive approach.

**Results:**

All participants felt that TB was an important issue in their community and that new detection strategies were needed. A “social-network strategy” was perceived as acceptable and feasible, where participants noted it was a caring act and could facilitate early diagnosis. For a “venue-based strategy,” most participants suspected TB transmission occurred at bars/taverns due to heavy alcohol use and prolonged time spent in crowded spaces; churches and betting halls were also commonly mentioned locations. Nearly all owners/leaders and patrons/attendees of bars, churches, and betting halls expressed acceptance of a venue-based strategy. They also indicated an interest in participating, citing many benefits, including increased TB knowledge/awareness, early diagnosis, convenience, and possibly reduced transmission, and recommended that the strategy incorporate sensitization, consent, volunteerism, and respectful, confidential, private services. For both strategies, most participants preferred the use of and being approached by trained peer TB survivors to facilitate ACF, given their prior TB patient experience and trust among community members.

**Conclusion:**

Stakeholders found social-network and venue-based TB-ACF strategies highly acceptable, recognizing their potential benefits for individuals and the broader community. Future research should evaluate the feasibility and effectiveness of TB ACF strategies for reaching non-household contacts.

## Background

Tuberculosis (TB) remains a major global health challenge, with TB ranking among the top 10 causes of death worldwide, driven by more than 10 million new cases each year (1). The World Health Organization’s (WHO) End TB strategy sets ambitious targets to drastically reduce TB incidence and mortality by 2035, which hinges critically on improving TB diagnosis (2). Despite progress, a massive diagnostic gap persists, with more than three million TB cases either undiagnosed or unreported yearly (1), highlighting an urgent need for effective and scalable case-finding strategies to identify and treat these missing cases and curb ongoing transmission in high-burden settings (3, 4).

Systematic screening for TB among individuals living in communities with a high prevalence is recommended by the WHO as an important control strategy (5). Modeling studies show that scale-up of interventions to identify persons with TB sooner is crucial to reducing TB incidence and associated mortality (6, 7). Community-based active case finding (ACF) may improve TB case detection by overcoming individuals’ key barriers to timely TB diagnosis and reaching those with limited access to health services (8, 9). However, there is mixed evidence for the effectiveness of different community-based ACF strategies, and systematic, community-wide TB screening is highly resource-intensive and is unlikely to be scalable in many low-resource, high-TB prevalence settings (3, 4, 10).

Given the social nature and airborne transmission of TB, focusing on contacts of persons with TB may represent a feasible and scalable approach to implementing community-based ACF in high TB prevalence settings. Historically, contact tracing has been a cornerstone of TB prevention and care efforts, focusing primarily on household contacts—many high TB-burden countries either are or have already implemented systematic ACF among household contacts (11, 12). While this represents an important strategy for reaching children and other vulnerable community members, emerging evidence suggests that 81–92% of TB transmission among adults occurs outside of the home in high-prevalence settings (13–16). Thus, extending contact tracing to non-household contacts of persons with TB may be a pragmatic first step toward community-wide ACF but has received little attention to date.

Using persons with current (i.e., receiving treatment) or former (i.e., completed treatment) TB (peers) to engage, screen, and educate non-household contacts is a promising implementation strategy. Many health programs in low-resource settings have implemented health services using peers to improve the uptake and availability of services (17–19). In the context of TB, former patients may serve as a credible source for healthcare experiences among peers and be more trusted than healthcare providers. Several studies from other high-burden countries in Africa and Asia suggest that peer-led TB community-based ACF strategies are feasible and acceptable (20–26); however, these studies largely focused on investigating household and/or known social contacts (20, 22, 24–26), which may have limited impact on TB transmission (14, 27) or community-wide door-to-door screening that resulted in a low TB case yield despite large numbers screened (21). To date, ACF strategies in high TB burden settings have not focused on casual contacts at community transmission venues (i.e., ‘hot spots’), which is where most adult TB transmission likely occurs (27, 28).

This qualitative study among several stakeholder groups in Lusaka, Zambia, sought to determine how best to implement contact tracing among non-household contacts. It explored the anticipated acceptability of and preferences for the delivery of two different community-based TB ACF strategies: (1) a “social-network-based” strategy where persons with newly diagnosed TB inform their *close contacts* within their social circles, including friends, family, and colleagues, and (2) a “venue-based” strategy, designed to identify *casual contacts*—those who may have interacted with individuals with newly diagnosed TB in crowded indoor community venues such as bars, churches, and minibuses. The “social-network-based” strategy, successfully used in public health and outreach programs, engages key individuals or “seeds” within a social network who can then influence their peers, friends, or family members to participate in a health intervention, access services, or spread information, due to their trusted, familiar connections (29). Meanwhile, the venue-based strategy, engages individuals in environments where they already feel comfortable, facilitating easier access to services or information without requiring them to be socially connected, but rather just to be regular users of select venues (30–32). We also specifically aimed to evaluate the anticipated acceptability of using trained TB survivors as peer community contact tracers among key stakeholders.

## Methods

### Study design and participants

We conducted a qualitative study using semi-structured in-depth interviews (IDIs) to explore individual experiences, preferences, and perceptions about TB transmission and screening strategies and gain insight into real-world feasibility and stakeholder engagement of social network-based and venue-based TB case finding strategies ahead of implementation in Zambia.

The study was conducted at the George and Matero public health facilities, and their catchment communities within Lusaka, Zambia; both communities have a very high TB burden, with 787 and 1,341 TB notifications in 2023, respectively.

Five stakeholder groups were sampled: We took a convenience sample of (1) formal healthcare workers who provide TB care/services and (2) informal healthcare workers (volunteers or peer supporters) who provide TB care/services at the study sites, and (3) purposefully selected persons with newly diagnosed TB (within the past 4 weeks) presenting at the clinic; among the two groups selected from the community, we purposefully sampled (4) owners and leaders at community venues where TB transmission was most suspected to occur by stakeholders, and conveniently sampled (5) community members without a current TB diagnosis (self-reported) who frequently attend such venues. Inclusion criteria included belonging to one of the five above stakeholder groups, being aged ≥18 years old, and providing informed consent. Recruitment was closed for each stakeholder type once information saturation was reached.

### Ethics, consents, and permissions

All participants provided written informed consent in their preferred language (Nyanja, Bemba or English). This study was reviewed and approved by The University of Zambia Biomedical Research Ethics Committee (#2292-2021) and the institutional review board of the University of California, San Francisco (#21-33701). The National Health Research Authority-Zambia gave authority to conduct research. The Provincial and District Health Offices and facility-in-charges gave permission to access the clinic. Owners and managers at the various venues gave their permission to approach staff and customers/attendees.

### Data collection

All data was collected over a two-month period between June and July 2022. This was after the peak of the COVID-19 pandemic but amidst continued low transmission rates in Zambia. Data collection was carried out in two phases. The first phase was conducted at the health facilities and involved semi-structured, in-depth, in-person interviews (IDIs) among persons with TB, healthcare workers, and TB peers/supporters. With permission of the facility-in-charge, the Research Assistants (RAs) directly approached healthcare workers found in the TB department about their interest in participation. Persons with TB presenting at the clinic were first engaged by a facility TB peer/supporter to introduce the study and assess interest in participation; if interested, a RA approached them to provide complete information on the study. All interviews were conducted in a quiet, private office space at the health facility.

The second phase of the study was conducted at community venues most suspected of TB transmission (‘hot-spots’) according to participants in the first phase, namely bars/taverns, betting halls, and churches. Bars/taverns were oversampled for recruitment as they were almost universally mentioned as a potential hot-spots. In-person IDIs were conducted among venue owners/leaders and attendees/patrons. The study team worked with trusted local Neighborhood Health Committee (NHC) members, who helped engage and introduce the study team to venue owners/leaders who were interested in participating. After permission had been obtained from the venue owner/leader, community members at each venue type were approached discreetly by a study team member about participation. IDIs were carried out during less busy times (mornings for bars/taverns and betting halls before their legal opening times, and weekdays for churches). To maintain confidentiality, IDIs were conducted in quiet, private, unused spaces within each venue or at nearby locations, and no personal identifiers were used during IDIs.

The data collection team consisted of three Zambian men and two Zambian women with at least a Bachelor’s Degree, who were highly experienced in qualitative data collection. While they grew up in similar environments, their education and training may have influenced data collection, analysis, and/or interpretation. We took several steps to minimize this influence. We trained our qualitative data collectors to be reflexive about their socio-economic and education status, which could be higher than some of the respondents. Data collectors dressed modestly, spent time building rapport and putting participants at ease, and used local parlance. Data analyses included the etic view of ADK and the emic view of CM, and all authors interrogated data and its interpretation, which was also discussed with the participating health facilities, their NHCs and community venue owners and leaders.

Interview topics for all stakeholder groups included TB-related risk perception based on the concepts of perceived severity and susceptibility in the health belief model (33). Interviews probed on TB being a health issue in the community, perceived risk of getting TB either by self or in the community, and suspicions about how and where TB transmission occurred in their community (Supplementary material); interviews also probed the perceived acceptability of, preferences for, and recommendations to improve the two primary “social-network-based” and “venue-based” strategies described below for reaching and screening non-household contact persons for TB in their community (Table 1) (34, 35).

**Table 1:** Descriptions and key design considerations for two potential strategies for improving TB detection among non-household contacts of persons with TB in Lusaka, Zambia.

**“Social Network Strategy”** **Step 1.** Diagnose individuals with active TB disease (at the health facility or during community-based activities). **Step 2.** Ask individuals with newly diagnosed TB to list all ‘close’ contacts outside of their home (e.g., family, friends, peers) with whom they spent substantial time before being diagnosed. **Step 3.** Perform outreach to all named close contacts to: (a) notify them of their TB exposure, (b) recommend or perform TB screening (and further testing if indicated), and (c) provide brief TB-specific education. ***Design considerations and preferences explored with stakeholders:*** ***Who** should perform outreach to close contacts—individuals with newly diagnosed TB or healthcare workers? What type of healthcare worker—professional or trained peers (i.e., lay)? Who are the key stakeholders that should be involved in the design and implementation?* ***What** should be included in the outreach activities? Are incentives needed to motivate peer outreach? What information or training would be needed before undertaking peer outreach?* ***How** should outreach occur—face-to-face or SMS/phone-based?* ***Acceptability and feasibility:** Would individuals with newly diagnosed TB participate in naming contacts and/or reaching out to close contacts? Would individuals, if named as close contacts, accept being engaged in community outreach and screening activities? What are the advantages and disadvantages of, and potential barriers of such a strategy? What recommendations are there for improvement?*
**“Venue-based Strategy”** **Step 1.** Diagnose individuals with active TB disease (at the health facility or during community-based activities). **Step 2.** Ask individuals with newly diagnosed TB to list all community venues they frequently attended and/or spent substantial time at before diagnosis. **Step 3.** Perform outreach to individuals attending named community venues to: (a) notify them of their potential TB exposure, (b) recommend or perform TB screening (and further testing if indicated), and (c) provide brief TB-specific education. ***Design considerations and preferences explored with stakeholders:*** ***Who** should conduct outreach activities at named venues—formal healthcare workers, trained peers, or venue owners/leaders? Does age or gender matter? Who are the key stakeholders that should be involved in the design and implementation?* ***What** activities should occur before any outreach occurs? What should be included as part of any outreach activities? Are incentives important for participation?* ***When** should outreach activities occur—what time of day and what day of the week?* ***Where** should outreach activities occur—inside or outside the venue? Onsite or nearby?* ***How** should outreach occur—face-to-face or distributing paper-based contact cards? Should individuals or groups be approached?* ***Acceptability and Feasibility:** Would individuals with newly diagnosed TB participate in naming the venues they frequented? Would individuals at named community venues accept being engaged in community outreach and screening activities? Would owners/leaders allow case-finding activities to occur at their venues? What are the advantages and disadvantages of, and potential barriers to such a strategy? What recommendations are there for improvement?*

IDI guides were informed by the updated Consolidated Framework for Implementation Research (CFIR) (34), a comprehensive implementation science framework that outlines five key domains influencing implementation effectiveness (See Supplementary Table S1), to better understand the implementability of non-household contact tracing (35). The updated CFIR was selected for its systematic approach to assessing, guiding, and understanding the multifaceted aspects of implementation within complex settings (health systems and communities), ensuring that all critical factors influencing the adoption, implementation, and sustainability of the non-household contact tracing strategies were considered. In particular, the updated CFIR domains and constructs have been successfully used to recognize that implementation involves social processes intertwined with the operational context (29), which makes it a suitable framework to identify and mitigate interacting influences before implementation for conditions such as TB (36–39).

### Data analysis

All interviews were recorded with permission from participants, transcribed verbatim, and directly translated into English from Bemba or Nyanja if applicable. A rapid, structured, deductive approach was used for the qualitative analysis. First, each interviewer (MF, HN, ZB, MS) captured key concepts and questions of interest within 72 h of interview completion using a structured debrief form organized according to CFIR domains. A second reviewer (ADK) then assessed the accuracy and completeness of each debrief form using the interview transcripts and made any needed changes. The contents of all debrief forms were then entered into a data matrix in Microsoft Excel, with each row representing a participant and columns representing key domains captured within the debrief form. The data matrix was then analyzed according to each of the five stakeholder groups, and key concepts and data points were summarized by reading down each column across all participants. The results were organized according to key themes emerging from the data within each CFIR domain, i.e., inner and outer setting, characteristics of the individual, innovation characteristics, and implementation processes (34).

## Results

### Participant characteristics

Overall, 56 participants were recruited, including 10 professional healthcare workers, 10 lay healthcare workers, 12 persons with newly diagnosed TB, as well as 12 owners and leaders of bars/taverns, churches, and betting halls, and 12 community members who frequent such venues. The characteristics of participants are summarized in Table 2. No individuals declined to participate; however, one church leader did not permit recruitment of attendees to avoid giving the impression that a TB case had been identified at their venue.

**Table 2:** Participant characteristics.

	Overall (*n* = 56)	Persons with TB (*n* = 12)	Professional healthcare workers (*n* = 10)	Lay healthcare workers (*n* = 10)	Venue owners and leaders^b^ (*n* = 12)	Community members (*n* = 12)
Community						
George	25 (45)	6 (50)	3 (30)	4 (40)	6 (50)	6 (50)
Matero	31 (55)	6 (50)	7 (70)	6 (60)	6 (50)	6 (50)
Age						
18–29	8 (14)	2 (17)	1 (10)	1 (10)	3 (25)	1 (8)
30–49	28 (50)	5 (42)	5 (50)	2 (20)	7 (58)	9 (75)
≥50	20 (36)	5 (42)	4 (40)	7 (70)	2 (17)	2 (17)
Sex						
Male	26 (46)	11 (92)	0	0	7 (58)	8 (67)
Female	30 (54)	1 (8)	10 (100)	10 (100)	5 (42)	4 (33)
HIV status^a^						
Positive	10 (42)	6 (50)	–	–	–	4 (33)
Negative	14 (58)	6 (50)	–	–	–	8 (67)
Prior TB disease						
Yes	15 (27)	5 (42)	1 (10)	4 (40)	1 (8)	4 (33)
No	41 (73)	7 (58)	9 (90)	6 (60)	11 (92)	8 (67)
Community venue type						
Bar/tavern	12 (50)	–	–	–	6 (50)	6 (50)
Church	8 (33)	–	–	–	4 (33)	4 (33)
Betting halls	4 (17)	–	–	–	2 (17)	2 (17)

a HIV status not ascertained for healthcare workers and community venue owners/leaders.

b Bar leaders included owners and managers; betting hall leaders included owners and managers; church leaders included pastors, priests, bishops and elders.

### Inner and outer setting

#### The importance of TB within the community

When asked about the top 2 health issues facing their community, most participants mentioned TB, and when further probed about whether TB was an important issue in their community, all participants agreed, noting that it was highly prevalent, dangerous, and a *deadly disease* if not diagnosed and treated quickly. Many noted that they knew people with TB, including some who had suffered and died, as exemplified in the quote below.

*“Like us here in XX we face problems … here we have TB … TB is a problem because it is a dangerous disease because you cannot compare someone with AIDS to someone with TB. TB kills. No wonder I see it to be a problem, because if you delay starting treatment you will die. I have seen a lot of my friends die.” (Male, Bar goer).*

### The need for improved awareness and TB case-finding strategies

Among community participants, there was near universal consensus about the strong need to raise awareness about TB through sensitization activities.

*“… It’s really important because that is part of health, a healthy community should be TB free.” (Female, Churchgoer).*

Several participants, including a female bar leader, said, “There is no knowledge” among community members about TB despite its high local prevalence, and that misconceptions about how it is spread and incorrect information existed in the community about symptoms, and the importance of prevention, early diagnosis, and treatment. For instance, one male church leader remarked that*, “ignorance is expensive.”* Participants cited the need for community-based TB awareness and education because health education is *“limited to the lucky ones”* presenting at clinics (Female, Bar leader).

*“I think first of all start sensitizing us about TB. What is TB? We should not just know that it is TB. But what is TB, what causes TB? What are the benefits of getting screened? … It will be more appealing for someone … because sometimes we fear … We do not know what we are getting tested for. So, it is better you sell out the idea to people, tell them about TB and all those things, then it will be easy to appeal to people for them to go for testing.” (Female, Churchgoer).*

Several participants also stated that stigma and fear remain ongoing challenges.

*“… What I see here in XX, I have a lot of friends, some you could even see that his or her health is deteriorating … they do not want to visit health centres due to fear of being found with HIV and even TB.” (Male, Betting hall goer).*

To mitigate the stigma surrounding TB and improve health outcomes, it was recommended that TB should be discussed as often and openly as other public health concerns such as “*COVID, malaria, and polio*,” (Female, Churchgoer).

When asked directly, all participants said that new strategies were needed to find more persons with TB and to find them sooner in order to stop continued spread, because many people may have TB and not know they are exposing others. A few cited the large number of TB cases and related deaths in each community as evidence that TB detection was going undetected. Many participants highlighted the several barriers/challenges to TB diagnosis faced by community members, including a lack of awareness of the disease symptoms that can inadvertently contribute to delayed diagnosis and continued transmission, as well as difficulties accessing health facilities due to fear and the time and costs involved.

To overcome such barriers, community-based outreach activities were frequently recommended to meet people where they are, especially at community places where individuals gather (e.g., bars and churches). As part of such efforts, the need for widespread community TB sensitization came up repeatedly, including having persons go around with megaphones and also going door to door to raise awareness.

### Characteristics of individuals

#### Perceptions on how and where TB transmission occurs within the community

Most participants, including those recruited from the community, correctly described that TB spreads through the air by coughing in congested and poorly ventilated places. Several also highlighted what they perceived as associated factors, including heavy alcohol use, smoking, and HIV. While not risk factors for TB, sharing beer cups and the fact that TB ran in families (i.e., hereditary) were commonly mentioned, representing points of incorrect knowledge.

When asked directly where they felt TB transmission was occurring, nearly all participants across stakeholder groups said that bars/taverns represented locations where TB transmission was likely occurring, citing very crowded conditions where people spend many hours at a time, heavily use alcohol, share cups, and smoke.

*“The alcohol places hmmm hmm … For that one the council needs to look into it, especially the Chibuku ones [opaque beer taverns], they are very dangerous … A lot of people will be getting sick.” (Man with TB).*

Several persons with TB also thought that *“being found in bars and drinking beer … could be the cause …”* (Man with TB) of getting TB, implicating both the venue and behavior in TB transmission.

Several participants also cited churches and betting halls as possible hot spots, being places where people regularly gathered indoors and spent substantial time.

*“Especially in taverns [people can get TB] because that’s where people they are crowded… Even in a church, you know, when people come together, people they are crowded at one place, in church.” (Female, Churchgoer).*

Less frequently mentioned locations by participants included minibuses, nightclubs, workplaces via occupational exposures and hazards, schools, funerals, and densely populated compounds.

### TB risk perception

TB risk perception varied substantially by participant type. Notably, all persons with newly diagnosed TB said they were not worried about TB before their diagnosis. They said this was due to insufficient TB-related knowledge or because they simply did not feel at risk. Two participants said they were shocked to learn that it was TB that was making them feel ill.

Among community members, risk perception appeared to be associated with the venues from which participants were recruited. Most persons at bars were quite worried about getting TB, citing a prolonged time in close contact with many people and being around what some participants described as “*careless coughers*,” who coughed frequently without covering their mouths.

*“… I am very worried that I could have TB because I meet a lot of different people because sometimes you find that you are not putting on a face mask then you are in contact with someone who is coughing.” (Male, Bar goer).*

Two (of four) persons from betting halls were worried about getting TB, and only one churchgoer (of four) voiced any concern about the possibility of getting TB.

*“As the way it is, I can get TB now just because of going in the groups, … It’s my worry I can get TB, cause we breath different (air). I worry now how I can get TB even in church when we are singing the songs as a group, yeah.” (Male, Betting hall goer).*

Like community members, risk perception for TB varied greatly among leaders and owners of different venue types. For example, all participants from bars or betting halls were either somewhat or very concerned about the possibility of getting TB, since they mix with so many different people, who may not disclose their status, including one survivor who was very worried about getting TB again.

*“…Yes, I get worried because I serve so many people. So I cannot know because, as you said, TB is spread through the air. I am found by the counter—that’s why the boss here gives me face masks…” (Female, Bar leader).*

*“I worry very much (about TB)… I worry very much on that one because the number of people that come to our shop is [a] very huge number of people, so it’s very worrying to my colleagues there and to other people there and other customers who come to our shop.” (Male, Betting hall leader).*

For reasons similar to the above, including that many different customers spent prolonged amounts of time in crowded conditions at their venue, the majority of bar leaders were also very concerned about their patrons getting TB at their venue.

*“I do worry a lot [about patrons exposing other customers to TB] because I look at the type of people, those that are in this community.” (Female, Bar leader).*

*Interviewer: “When you open your bar, and the customers start coming in, do you ever feel worried that there might be someone with TB and might infect others or even you?”*

*Participant: “Those are things I see every day.” (Male, Bar leader).*

Church leaders had only minor concerns about getting TB themselves, noting that you cannot know who does and does not have TB—*“We are all human beings, you can get TB” (Female, Church leader)—*while others noted they are not worried because they know how to protect themselves.

Similarly, church leaders were also less worried about their congregation members getting TB. One noted it was because they have a program to support those who are sick (like with TB), while another said they discuss health very openly and encourage those who are sick to get help and support.

*“… Not very worried in a sense that we have a group that conducts health education during mass services when everyone is here, whereby we encourage them to say, ‘When you notice such and such, run’ [to the clinic]” (Male, Church leader).*

### Innovation characteristics

#### Perceptions toward a social-network-based TB case-finding strategy

The perceived acceptability and potential effectiveness of a social network-based TB case-finding strategy to reach more non-household contacts was explored among participants (Table 1). Formal and lay healthcare workers were skeptical about the strategy being acceptable to individuals with TB given community associations with HIV (and not wanting to be identified as having HIV), and more generally, a fear of being stigmatized for having TB; this was rooted in their years of observations of how stigma affected TB care engagement and medication adherence.

It was, therefore, notable that all persons with newly diagnosed TB said they would be willing to share their close, non-household contacts with healthcare workers. They said it would not be a problem because it is a trusted healthcare worker asking and that they would want those close to them also to get the help they need.

*“You want to help that friend [to find] out whether they have it not” (Woman with TB).*

Furthermore, those with newly diagnosed TB universally agreed that contacting their close, non-household contacts was a good idea, and all said they would be willing to participate. They said that they wanted to provide education, share their experience, and protect the health of those close to them—one participant said they had already reached and encouraged their close contacts. Others also noted that this was similar to what had already been done in their community for HIV and COVID-19.

*“I would accept this program, and I would love it …” (Man with TB).*

*“… I would also encourage them to get tested, I would tell them, ‘Let us go and get tested … We must go’.” (Man with TB).*

Several said it would feel good to help, with one participant drawing parallels with being a preacher:

*“It is a blessing from God because it is the same as doing God’s work so that others can be protected so that diseases and deaths are reduced in the country.” (Man with TB).*

A few participants noted that if they were in such a position, reaching out to non-family members could be difficult and that they would not be confident; therefore, it was recommended that persons with TB first receive brief education on how to approach people and about TB itself as part of social-network case finding strategy.

Most persons with TB felt that their close contacts would be grateful for the information and receive it well. However, a few did note that some persons could react poorly, especially as it may be perceived that they are implying that their contact has TB disease—they may even become hostile *and ask, “have you come to fight with me?” (Man with TB).*

When community participants were asked about being contacted by a friend or relative with TB, who would then share their experience and recommend TB screening/testing, all said that this was a good strategy and that it would be acceptable to them. Many said that it was “*an act of love*” that showed that the person cared for them and would help them stay safe and healthy because they are at risk after their TB exposure.

*“That person wants to save me. Others might have fear and even insult that person. As for me, I would not be scared; I would tell him, ‘You have done well, so let us go. You know what it feels like, and you want to protect me.’ So I would go.” (Female, Churchgoer).*

*“Those people, they care for me because someone who does not love me would have just let me die … I receive love from that person because they do not want me to go through what they have gone through.” (Male, Bar goer).*

This strategy was identified as advantageous because it could help get an earlier diagnosis and stop TB spread to their family, friends, and community.

*“Yes I can [accept it] … I have to know because maybe I am usually found with that person. Then they come and tell me that they found me with TB. So, I have to accept … Oh, let me go also check myself so that I can start treatment early.” (Male, Bar goer).*

No participant said that they would be upset with or blame the relative/friend/peer who informed them though they thought others might. Additionally, a few participants said that they would initially be upset by the information, but they would simply need time to process it.

### Preferences for implementing a social-network-based TB case finding

Community members and persons with TB were asked to imagine they were exposed to TB by a friend, relative, or colleague and then rank 4 different approaches for delivering a social-network strategy and to explain what drove their preferences (Table 3). This included (1) having their close contact reach out to them in person to notify them of their exposure and recommend they go to the health facility, (2) having their close contact give them a paper contact card with information about their exposure, brief TB education, and where to go if symptomatic, (3) having their close contact call them or send an SMS about their exposure, or (4) having a trained peer who was a TB survivor reach out to them in person to notify them of their exposure, provide education and conduct symptoms screening.

**Table 3:** Rankings of different delivery approaches for a social-network-based TB case-finding strategy to reach non-household contacts among community members and persons with TB.

Delivery approach	Overall rank	Aggregate rank by participant type		Rationale^a^
		Persons with TB (*n* = 12)	Venue patrons and attendees (*n* = 12)	
Told by a trained peer (TB survivor) from the facility	1	1	1	**+** Have first-hand knowledge of TB; can be trusted because they are trained and from the facility **+** Unlike their contact, a trained peer can offer on-the-spot screening and inform on the next steps **+** Unlike their contact, a trained peer is much better able to answer any questions
Told face-to-face by a relative/friend/peer with TB with whom they were in close contact	2	2	2	**+** They are well known to them, and it shows that they care for them **+** Telling them in person shows that they are serious about the information, and it carries more weight to motivate contact to get help fast
Given a paper contact card by a relative/friend/peer with TB with whom they were in close contact	3	3	3	**+** It is easy to read and act on advice; it can be trusted as it is from the facility **+** Could later serve as a reminder **−** Easier to ignore advice and throw it away
Receive an SMS or phone call from a relative/friend/peer with TB with whom they were in close contact	4	4	4	**−** May be perceived as a joke and is harder to trust **−** It is easier to ignore **−** Information may not reach them as many do not have phones

a “**+**” indicates a positive feature of the delivery approach; “−” indicates a negative feature of the delivery approach.

While nearly all approaches were acceptable to individuals with TB, there was greater heterogeneity among community members as to what would be acceptable. Overall, being told of their TB exposure by a trained peer, as opposed to their close contact, was the most acceptable approach; several positive aspects were mentioned, including their first-hand knowledge of the disease, their trustworthiness as a health facility representative, and the convenience since they can provide symptoms-based screening immediately (and refer if indicated) and answer any questions.

*“I will not have thoughts of doubt but will follow what that person tells me because that person is … I can say he/she is a health worker because he/she knows this disease. Yes, and what to follow in order for someone to be cured.” (Man with TB).*

*“The reason is because when one introduces himself as one who works at a known place, it gives us confidence because if there is one person who can destroy anything, it is the person who is trained, because people trust the trained people and we cannot even argue.” (Male, Betting hall goer).*

The second most preferred approach was being informed face-to-face about their exposure by their close contact, as they are well-known to that person, it demonstrates that it is serious, and that they cared for them.

*“… Maybe they have seen my health is not okay and they advised me to get tested.” (Man with TB).*

*“I think it is easier than telling maybe the patients to leave numbers of people close to them, I think that may be a bit tedious for people working in that facility.” (Female, Churchgoer).*

The third most preferred option was to receive a paper card from their close contact, that provided basic TB education and information about where to present if they had any current symptoms; participants felt this would be easy to read and trustworthy because it came from the facility, but also highlighted the negatives that it was easy to just ignore and/or throw away. The least acceptable option was to receive an SMS or phone call from their close contact—especially an SMS; this was felt to be harder to trust/believe, including potentially a joke since they are not coming to them directly with this serious information; they preferred to be told in person to emphasize the importance of the message and avoid making a person feel mocked. It was also mentioned that some persons do not have phones, and if they do, you may not know if an SMS ever reached them with the information. More than half of community members and a few persons with TB would not find this approach acceptable.

*“The phone looks like you are mocking someone. Why did you not tell me just there and then, you are calling me after I have reached home?” (Man with TB).*

### Perceptions toward a venue-based TB case finding strategy

The acceptability and potential effectiveness of a venue-based TB case-finding strategy (Table 1) was explored among venue owners/leaders and patrons/attendees across bars, churches, and betting halls. Participants across both groups almost universally accepted the strategy, with leaders/owners saying they would allow TB outreach and screening at their venue and patrons/attendees saying they would participate in such activities. Both groups shared a common desire for health protection—most said that they would want to know their TB status if exposed and that it would help them and others to get the help they need sooner. Thus, most participants felt it would be an effective strategy.

*“I would want to protect myself and others. My colleagues, I would want them protected because I would not feel good to know that someone got TB from here. It would be very bad, even me, what if I got it from work? So, I think it can be a good thing and all our customers can agree if someone came to test them. I think that its a very good idea so that we can all be protected …” (Female, Bar leader).*

*“Yeah, for me personally, I would still accept because of the exposure. So, if people have been identified to say, ‘These people were exposed, They’ve got TB, can you also go for testing?’ That can be an option that I can take.” (Female, Churchgoer).*

*“The benefits would be, I get diagnosed and I check myself now before it’s too late to start getting treatment … The earlier the better.” (Male, Bar goer).*

Participants in both groups valued the strategy for its convenience by bringing health services to community gathering places, making TB screening more accessible and potentially increasing community awareness and reducing transmission.

*“It’s like bringing things … uhmm health services closer to where people are. You do not need to walk from here. Just here, and it will be easy for the health personnel to find these people, because we’ll be all here.” (Male, Churchleader).*

*“The benefits would be being helped, healed, and others would actually know that they can even go to the clinic to get tested. If you are not coming, no customer would actually know that they can be helped in the bar ….” (Bar owner).*

*“What would make me accept [TB screening at the bar] more is that I do not have time to go to the health facility … I would not want to go to the clinic and test but rather do it at the bar whilst I am drinking so that I know [TB status].” (Male, Bar goer).*

Another participant highlighted the potential of the strategy to reach many individuals at once, including those who may be reluctant to get screened for TB.

*“Everyone will be around and no one will run away … [Otherwise], If they say that we should, you will find that out of a thousand people, only four hundred would show up, and the six hundred would nowhere to be seen, which means that the disease will not reduce … It will only be increasing.” (Male, Churchgoer).*

A few participants said that the venue-based TB case finding strategy was acceptable, because it was already done in their venue for HIV and COVID-19.

*“Nothing needs to be done [to improve acceptability] because it is about saving lives. Just like they welcome staff for HIV and COVID that’s how we can welcome you also.” (Female, Bar leader).*

Both groups also highlighted the broader community benefits, such as helping to raise awareness and possibly reducing transmission.

Concerns about the venue-based TB case-finding strategy were infrequent but notable. Both leaders/owners and patrons/attendees expressed apprehensions regarding the potential effects of TB-related stigma. They shared a reluctance to be approached for TB screening in public settings, such as bars or betting halls, fearing rumors and embarrassment. Additionally, being contacted by an unidentified “stranger,” especially if not clearly identified as from the health facility, raised the possibility of defensive or hostile reactions from individuals. Only one bar owner specifically feared the impact of such activities on their business.

### Preferences for designing a venue-based TB case finding strategy

Preferences for several design-related considerations were explored with community venue owners/leaders, patrons/attendees of such venues, and persons with TB. Most participants said the characteristics of the person conducting outreach and screening activities, such as age, sex, and whether they were a professional healthcare worker did not matter, as long as they possessed certain aspects of professionalism. For example, when directly queried about using a trained peer vs. a professional healthcare worker, most participants said what mattered to them was that they were a representative of the health facility (and could show ID), were trained to do their job, could answer questions and provide good information, and were polite and respectful. A few expressed a clear preference for a professional healthcare worker given their experience and maturity, while one felt a trained peer’s experience surviving TB could be motivating and inspiring. A couple of bar leaders/employees voiced a preference for a man due to what was described as the “*nature of bars*.” Also, a small number of participants indicated a slight preference for older persons as they were more likely to be mature and taken seriously.

The importance of the day of the week and time of day for conducting screening activities was also assessed. Venue owners/leaders found nearly any day of the week and time of day acceptable for conducting outreach and screening activities, provided (and most importantly) permissions were agreed to and secured in advance. For potential days of the week for activities, weekends were most frequently mentioned because that is when they were busiest. Only two owners/leaders mentioned weekdays as being logistically easier due to fewer people to screen, and only one mentioned potential revenue loss by selecting a busy weekend day. Among patrons/attendees, weekends were strongly preferred by all because they were not working and were thus more available, but most said they could accept almost any day that was offered.

Among venue owners/leaders, the most frequently recommended time of the day was when things were busier at their venues—early/late afternoon in bars/betting halls, cautioning that any later, and patrons may be inebriated and, in churches later morning after service. Patrons and attendees almost universally said they could accept screening anytime, but a few said in the morning; none preferred late afternoon or evening.

Venue owners/leaders and patrons/attendees were asked to consider and rank three specific delivery approaches for a TB case-finding strategy to hypothetically reach patrons/attendees who may have been exposed to a person with TB at that venue. These included (1) having a trained peer (TB survivor) conduct outreach and screening activities, (2) providing the venue staff education and training to have them screen their own patrons/attendees, and (3) having venue employees hand out a paper contact card with information about their potential exposure, brief TB education, and where to go if symptomatic (Table 4).

**Table 4:** Rankings of different delivery approaches for a venue-based TB case-finding strategy to reach non-household contacts among community venue owners/leaders and patrons/attendees.

Delivery approach	Overall rank	Aggregate rank by participant type			Rationale^a^
		Venue owners and leaders (*n* = 12)	Venue patrons and attendees (*n* = 12)	Persons with TB (*n* = 12)	
Trained peer conducts outreach and screening	1	2	1	1	**+** They come from the health facility and are trained; they are competent and trustworthy **+** They can answer questions **+** Their experience as a TB survivor will be engaging and motivating
Hand out paper contact card to patrons/attendees	2	1	2	2	**+** They are easy to hand out at venues. **+** May reach those who are too scared to accept venue-based screening **+** People can ‘take it or leave it’ and decide privately what they want to do **+** Could be shared with friends/relatives − May be easy to ignore/throw away. − Some persons cannot read
Venue staff is trained to screen patrons/attendees	3	3	3	3	**+** Know patrons/attendees well; know how to talk with them and are respected (“their place”) **+** Easy to reach many people as they are already serving them − Patrons may be suspicious; may lose business − Staff may be too busy to conduct training − May not be well trained; unable to answer questions

a “**+**” indicates a positive feature of the delivery approach; “−” indicates a negative feature of the delivery approach.

The approach using trained peers—specifically TB survivors—for outreach and screening was the most favored overall approach, being most preferred by church leaders, venue patrons/attendees and persons with TB. This approach resonated strongly due to the peers’ association with health facilities, which conferred trustworthiness, and their personal experience with the disease, which provided a compelling narrative for education and motivation.

*“Because the survivor … They say ‘pain is understood by the one experiencing it,’ he knows what happens, he knows how scared he was, he knows how he received help. He is a person who knows how to penetrate into the mind of this person and be able to help that person out of the problem.” (Male, Church leader).*

*“I will not have thoughts of doubt but will follow what that person tells me because that person is … I can say he/she is a health worker because he/she knows this disease. Yes, and what to follow in order for someone to be cured.” (Man with TB).*

*“Because he has gone through, he knows where you are going. He has seen that and gone through it, so let us go and go this way.” (Man with TB).*

Participants especially valued the authentic connection and relatability that survivors could offer, with church leaders seeing parallels with testimonial practices in their congregations, underscoring the effectiveness of personal stories in inspiring action.

*“I think it can be very effective because someone is speaking as an informed person, someone who has experience. And given that they are sharing their experience so that, people are able to gain more interest because you are not just talking from theoretical understanding, but it is something that you have undergone. I have seen that also to be very, very effective. That’s the same like we use in the church like the person is going to share their testimony and yeah, faith …” (Male, Church leader).*

The distribution of paper contact cards was also highly acceptable to participants for its straightforward and non-intrusive nature. Bar and betting hall leaders ranked it their top choice due to its simplicity and allowing individuals the autonomy to follow up on their potential exposure.

*“It will be very much easy. It will just be like, ‘Oh guys, here, these are the papers. If anyone is experiencing the symptoms that are listed in those papers, go for TB screening.’ So that will be very much easier.” (Male, Betting hall leader).*

Patrons and attendees acknowledged this method’s convenience and the potential for broad dissemination, including their ability to be shared with friends; however, they also expressed the necessity for additional context to be provided with the cards as to why they were being handed out, in order to enhance their impact and ensure they were not misunderstood or simply thrown away. It was also highlighted that the effectiveness could be limited among participants who cannot read.

*“… It is also a notice that they are giving us. They are announcing, ‘So if you are seeing that on your body, go to xxx clinic to get more information.’ That is good.” (Male, Betting hall goer).*

*“People should know the benefits. People should know why they need to be screened. So that can also be a good strategy so long they do not just dish out the papers. As long as you explain to someone ‘We know there is TB, we know there is this, so one of us maybe was exposed. We would encourage you to go for testing.’ That can be a good option, so long as the people that you give the papers are able to explain.” (Female, Church goer).*

The least preferred delivery approach by all participants was having the venue staff receive training to conduct TB screening. While it was highlighted that the staff knew their patrons/attendees well, were largely respected by them, and could easily reach them when serving them at their venue, it was also noted that patrons/attendees could be suspicious of their intentions, possibly resulting in lost business due to fear of TB testing. Venue attendees/patrons also voiced concerns about their lack of training in healthcare, what the staff intentions were, and the public nature of TB screening.

*“People might stop coming … Because they will be saying that when they come here they will be tested. People are difficult, you see. Not knowing that you just want to help them, you see.” (Female, Bar leader).*

*“He is the owner, but some things would not look nice being done in public.” (Male, Bar goer).*

Pragmatically, venue owners/leaders also said they/their employees may be too busy to try to conduct any form of TB screening. For these reasons, several participants across all groups said they would not participate in such a program if it were implemented.

Despite the overall high acceptability of a venue-based strategy across participant groups, several recommendations were provided to maximize the acceptability, regardless of the delivery approaches utilized (Table 5). Across all groups, the strongest and most consistent recommendation was that patrons/attendees should be treated with kindness and respect. Additionally, many participants emphasized that all activities should be conducted outside the venue—specifically, personnel should refrain from entering the establishment, with all operations taking place in open areas immediately adjacent to or in the vicinity of the premises. Several recommendations centered around activities that should be conducted in advance of any screening activities, including obtaining approvals from owners/leaders, advertising the dates/times of TB screening activities, and first providing education to help people understand the benefits of early diagnosis and treatment, which could help to generate demand for services. During any screening activities, it was suggested that the rationale for conducting TB screening should be made clear, that the confidentiality of results should be ensured, and that the voluntary nature of participation should be emphasized. Patrons/attendees and persons with TB consistently recommended healthcare workers have identification visible at all times, while one said not to use fear-based language but instead explain the risks and benefits Other recommendations included approaching groups of individuals about participation in TB screening activities rather than any one individual so they do not feel singled out and to put up portable tents outside of the venue to establish privacy.

**Table 5:** Recommendations for improving the acceptability of a venue-based TB case finding activity to reach non-household contacts.

Recommendation	Venue owners and leaders (*n* = 12)	Venue patrons and attendees (*n* = 12)	Persons with TB (*n* = 12)
Get approvals from owners/leaders in advance	✓		
Advertise screening activities in advance to create awareness	✓		
Provide TB education in advance of screening activities, make people aware of benefits of early diagnosis to create demand	✓	✓	✓
Clearly identify as a healthcare worker from the facility and have identification visible		✓	✓
Approach should be thoughtful*—*use kind, respectful, and supportive language	✓	✓	✓
Approach entire groups so individuals do not feel singled out		✓	✓
Clearly state why you are approaching them*—*provide a clear rationale for why TB screening is being conducted	✓	✓	✓
Conduct activities outside the venue—avoid any activities in the bar/betting hall itself.	✓	✓	✓
Emphasize the benefits and risks, but do not be fear-based		✓	
Let persons voluntarily decide to participate	✓		✓
Emphasize and ensure privacy and confidentiality	✓	✓	✓
Set up tent(s) outside the venue for privacy.	✓		✓

“✓” indicates that the suggestion was made by the respective stakeholder group.

### The willingness of persons with TB to serve as “TB ambassadors”

Interest in serving as a peer TB ambassador to facilitate community-based non-household contact tracing (social-network based and venue-based) through sensitization and screening was explored among persons with TB. They were told they would be provided brief, standardized training and education and provided a financial stipend. Nearly all said they would be willing to serve as TB ambassadors and felt it was a good idea. They would want to help others to prevent them from experiencing what they had experienced and felt they could do a good job reaching others and providing them with education and support. A few also highlighted it would be a good employment opportunity, and that the stipend could be very helpful, since TB survivors often find themselves unemployed. Some also explicitly said that TB survivors were best positioned to do such work.

*“I would be very thankful because I would be helping people’s health. It would be a very good thing …” (Man with TB).*

*“[I would want to participate] because I would not want them to go through the problems that I have seen … Because if I help someone, they also will go and help someone else, who will go and help someone else, so that will be the case, and as a result, you will find that the TB cases are going down.” (Man with TB).*

Two participants noted the logistical challenge of becoming a TB ambassador due to their current work obligations making such a commitment difficult; however, one said they would happily quit their job to be an ambassador if the program were to become available, because it would be easier than their current job as a construction worker and they would enjoy helping others.

### Key stakeholders to engage for successful implementation

Participants across all groups named many different stakeholders who were important for engaging in the design and implementation of a community-based strategy to identify non-household contacts, especially at community venues. The most mentioned were community venue owners and leaders, including bar owners, pastors and elders, shop owners, market chairpersons, and bus station chairpersons. Many also mentioned other influential community members, such as water tap leaders, local ward leaders and council persons. Many said that the involvement of the NHC members and professional and lay healthcare workers was crucial. The National TB Program was also mentioned as a key stakeholder for the implementation and sustainability of any novel case-finding strategies in the context of existing control efforts.

## Discussion

Participants from five diverse stakeholder groups from two Lusaka communities unanimously recognized the high prevalence and risks posed by TB in their communities, underscoring the urgent need for innovative strategies to improve its detection. The study revealed very high acceptability for two novel strategies to reach often overlooked non-household contact persons—a “social network-based” and a “venue-based” strategy—were valued for their community-centric approach that meets people where they are, promoting ease and convenience. Participants indicated a strong willingness to participate in both strategies, reflecting their potential to significantly enhance TB case finding. However, the success of these strategies will hinge on thoughtful design and implementation, taking stakeholder preferences into account, such as employing trained TB survivors for outreach and ensuring all interactions are conducted with kindness and respect. Notably, despite TB being recognized as a top community health issue, participants suspected large gaps in community TB-specific knowledge, including symptoms and the benefits of early diagnosis, indicating that community sensitization and education activities must be included in any future community-based TB case-finding strategy to ensure a holistic and effective approach to reduce TB.

Our study assessed venues for tracing non-household contact persons and found that alcohol-serving venues, particularly bars and taverns, are perceived as high-risk TB transmission settings, which is corroborated by several prior studies, including those using molecular epidemiology and social network analysis (40–44). In addition to their propensity for overcrowding, inadequate ventilation, and frequent, prolonged time spent in such venues, facilitating transmission, participants also correctly highlighted how heavy alcohol consumption—and typically concomitant smoking—directly increases the risk of TB disease after exposure (45–52). Although churches were also recognized as potential transmission sites due to their social importance where many Zambians spend prolonged time indoors together, the risk was deemed much lower than the above venues. This suggests that venue-based TB detection strategies must tackle both behavioral (e.g., alcohol reduction and cessation support) and locational risk factors to reduce TB and protect public health.

The high acceptability and potential effectiveness of a social-network-based strategy, which was perceived as a caring act in our settings, are further supported by a randomized trial in India among more than 3,000 persons with TB, which demonstrated that peer outreach was more effective in increasing TB screening and testing than outreach methods by healthcare workers, particularly when combined with a small financial incentive (24). Furthermore, a study conducted in Nepal found that volunteers living with HIV, when provided with brief training, effectively screened and referred others within their social network who also have HIV, for TB testing; this initiative was highly acceptable and led to a significant increase in new TB diagnoses (22). These results underscore the potential value of incorporating social networking strategies into efforts to improve TB detection efforts.

Additionally, there was substantial support among community members for a venue-based TB case-finding strategy. These findings sharply contrast with a previous study in Vietnam that undertook case finding among contacts of persons with multi-drug resistant TB (MDR-TB), with only 1 out of 17 locations agreeing to participate in contact tracing (53). In our study, notwithstanding stakeholders’ strong willingness to participate, some concerns about TB-related stigma and the public nature of screenings were raised, suggesting the need for designs that prioritize privacy and minimize potential social repercussions. To optimize the acceptability of venue-based TB strategies, stakeholders underscored several logistical considerations, including advance preparations, such as securing venue owner/leader approvals, advertising screening events, and providing education on TB and the benefits of early diagnosis. Participants almost universally agreed on the critical importance of clear, kind, and respectful communication for all encounters with patrons/attendees and owners/leaders in addition to other key considerations, including assurance of result confidentiality, emphasizing the voluntary nature of participation, conducting activities outside of venue, and the visible identification for healthcare workers. Simple paper contact cards, which have been previously demonstrated success in South Africa and Thailand, were also highly acceptable in the context of both novel strategies (54, 55). These cards could serve as a valuable adjunct to peer-led strategies, facilitating improved contact tracing.

In our study, stakeholders from diverse groups unequivocally favored the involvement of TB survivors in venue-based TB outreach and screening, valuing their credible, firsthand experiences when connecting with peers. This preference was bolstered by the high acceptability of engaging trained peers to extend social networking strategies to close, non-household contacts. Such findings are in line with research from high-burden settings like Cambodia and the Democratic Republic of Congo, where TB survivor-led initiatives have proven acceptable, feasible, and effective in improving TB detection (20, 21). Participants favored TB survivors for their empathetic engagement, trustworthy representation of health facilities, and the inspiration they offer as living proof of successful TB treatment, all of which motivates increased TB screening. Furthermore, our study revealed a marked willingness among individuals with TB to serve as ‘TB ambassadors’ in future community-based TB initiatives, underscoring a collective determination to actively participate in the TB response and to provide support to peers on their path to TB detection and treatment.

The study underscores the vital role that an array of stakeholders plays in the potential effectiveness of community-based TB case-finding. Participants particularly highlighted the importance of involving key community figures during the planning and implementation phases. Extending beyond healthcare workers and policymakers, such as those in National TB Programs, the inclusion of venue owners and leaders, local influencers, and civil society members was felt to be crucial. The WHO’s recent guidance supports this view, emphasizing the importance of community and civil society engagement in efforts to end TB, especially in TB sensitization activities (56). By tapping into the unique perspectives and intrinsic strengths of these varied stakeholders, TB case-finding strategies can be tailored to meet the community’s specific needs. This approach not only facilitates acceptance but also ensures smooth integration into existing TB prevention, diagnosis, and care measures, enhancing sustainability and reaching those who are most vulnerable and at risk.

A key strength of our study is the enrollment of a large number of participants from two high TB burden communities, representative of five diverse stakeholder groups from healthcare facilities and the broader community. This inclusive approach not only captured a broad spectrum of insights and preferences but also identified common ground and distinct outlier views. The depth and variety of stakeholder input gathered are pivotal in shaping TB case finding strategies that are grounded in the values and preferences of those that such strategies aim to reach and the insights from stakeholders essential to future implementation success. However, more men than women with lived experience were enrolled, limiting our ability to detect gendered differences in venues to prioritize for TB screening and safety concerns about revealing TB status to contacts in their social network. Another limitation of the study is the reliance on stakeholder perceptions regarding TB transmission venues, which may introduce subjective biases. Nonetheless, the high degree of suspicion around bars/taverns in particular, is aligned with prior data from more robust study design types, such as social network and contact pattern surveys or molecular epidemiological data (41, 43, 44). Additionally, social desirability on interview responses of hypothetical scenarios may overestimate the acceptability (and underestimate TB-related stigma) of the social networking-based and venue-based case-finding strategies, resulting in lower than anticipated participation (i.e., adoption and reach) upon implementation. Nevertheless, participants provided a wealth of persuasive and consistent reasons along with detailed explanations that not only reinforce their stated willingness to participate but also suggest genuine interest and perceived value in the strategies despite.

In conclusion, this study in Lusaka, Zambia, found almost universal anticipated acceptability among key stakeholders for both social networking-based and venue-based TB case-finding strategies to improve TB detection among non-household contacts. While the potential effectiveness (e.g., case detection yield) of such strategies is not yet understood, the groundwork laid by this research indicates a readiness within the community to adopt innovative community-based strategies for TB detection. Implementation research is needed to confirm the strategies’ acceptability while assessing their feasibility, potential effectiveness, and cost-effectiveness, both as standalone strategies and in comparison with existing TB detection methods, to ensure that the most preferred and effective strategies are implemented in high TB burden settings.

## Data Availability

The datasets presented in this article are not readily available due to the sensitive nature of the qualitative data collected for this study. This is to protect participant privacy. However, data that support the findings of this study, including detailed quotations, are included within the article itself to the fullest extent possible. Requests to access the datasets should be directed to Andrew Kerkhoff, Andrew.Kerkhoff@ucsf.edu.
