# Supplementary material for: Designing community-based strategies to reach non-household contacts of people with tuberculosis in Lusaka, Zambia: a rapid qualitative study among key stakeholders
Source: Front Public Health. 2025 Jan 13;12:1408213. doi: 10.3389/fpubh.2024.1408213 (PMC11769986; doi:10.3389/fpubh.2024.1408213)
Supplement: Supplementary file 1 [file Table_1.pdf]

Supplementary Table. Categories and themes emerging under CFIR domains and constructs

Domains	Constructs	Categories	Themes
Inner & Outer Setting	Socio-Cultural	Relative Priority	• TB is among top two health issues in the community<br>• TB is deadly<br>• Early detection can prevent death
		Community Priorities	• Need to raise awareness and disease recognition<br>• Need to correct misconceptions<br>• Need to address community stigma and fear
		Case-finding is a priority	• Early detection necessary to prevent spread<br>• Delayed care-seeking due to lack of disease recognition<br>• Cost and time at clinic also delay care-seeking
Characteristics of individuals	Need	Deficits in TB control	• Air-borne transmission makes most people at risk<br>• Alcohol-use, smoking, HIV as TB risk factors<br>• Crowded areas where people congregate<br>• Bar owners and workers feel at risk of TB
	Capability	Knowledge and control	• Church leaders and goers felt they knew how to protect themselves<br>• Bars/Taverns bring together strangers, increasing risk of transmission
	Opportunity	Power to prevent transmission	• Bar owners want to prevent TB spread to their clients and workers<br>• Church leaders urge congregation to seek care for TB symptoms
Innovation characteristics & processes	Assessing Needs	Innovation deliverers	• Healthcare workers thought people with TB would not deliver the strategy to non-household contacts<br>• Persons with TB willing to provide contact names or talk to contacts, provided they are coached<br>• Persons with TB willing to be ambassadors<br>• Owners unwilling to screen patrons themselves
		Innovation receivers	• Would be receptive to both strategies<br>• Some may think they are being accused of being the source of TB transmission<br>• Some may be upset with the message (exposed to TB) but never with the messenger
		Preference	• Community members preferred being reached by a trained peer<br>• Though second choice, they were open to being informed by their close contact<br>• Simply being given a paper card was acceptable, but easy to dismiss<br>• SMS prompts or trained owners/staff were not acceptable to many<br>• Owners and patrons were receptive to venue-based screening<br>• Venue-based screening has many advantages, including convenience<br>• Venue-based screening could be stigmatising, suspicious, and give rise to rumors (minor concern)<br>• Professional identification more important than gender or age of person doing outreach<br>• Owners prefer weekday and patrons weekend delivery<br>• Delivery should be earlier in the day at bars and later in the day at churches
