# Supplementary material for: Designing community-based strategies to reach non-household contacts of people with tuberculosis in Lusaka, Zambia: a rapid qualitative study among key stakeholders
Source: Front Public Health. 2025 Jan 13;12:1408213. doi: 10.3389/fpubh.2024.1408213 (PMC11769986; doi:10.3389/fpubh.2024.1408213)
Supplement: Supplementary file 2 [file Data_Sheet_1.PDF]

Peer-to-peer TB study (P2PTB)

TB Patients In-Depth Interview (IDI) Guide

Introduction

- How long have you lived in George/Matero?
- Tell me a little bit your daily activities before your TB illness?
  - If applicable: How were these activities affected by your illness?

TB Risk Perception and Acquisition

- Before you were diagnosed with TB, were you worried that you could get TB?
  - What makes you say that?
- How do you think you may have contracted TB?
  - Is there a <u>place/location</u> in the community where you think you may have been exposed to TB?
  - What makes you say that?

Preferences for and concerns related to non-household contact tracing

Prompt/transition: I'd like to now transition to ask you about some potential strategies for improving TB diagnosis in George/Matero. For each of the below scenarios, I would like for you to think back to before you were diagnosed with TB.

- First, imagine that you were in contact with a colleague, friend or family member that had recently been diagnosed with TB. What sorts of thoughts and feelings would you have had?
  - Can you tell me more about that?
- Now, imagine that that person (colleague, friend or family member) directly reached out to you to inform you that they had been diagnosed with TB and recommended that you get tested for TB if you had any signs or symptoms of TB.
  - How would you feel about being contacted by that person directly?
    - What makes you say that?
  - What would be your biggest concern about being contacted by that person directly?
    - What else?
  - What do you think the benefits might be, of having a colleague, friend or family member reach out to you directly to inform of their TB diagnosis and recommend you get tested for TB if you have any signs or symptoms of TB?
    - What else?
  - Would this strategy [have been] be acceptable to you?
    - What makes you say that?
- What sorts of things would make [would have made] this strategy more acceptable to you?
  - What else?

- Would it make [have made] a difference whether your colleague, friend or family member did this face-to-face, or via phone/SMS?
    - What makes you say that?
- Next, imagine that you were exposed to TB somewhere outside of your home, by someone you did not know (for example, at a bar, shop, or in a minibus). Now imagine, if someone you didn't know approached you somewhere in the community (for example at a shop, at a bar, or at a minibus stand), to inform that you had recently been in contact with someone with TB and screened you for signs and symptoms of TB.
    - What would be your biggest concern about that?
        - What else?
    - What specific concerns would you have about this interaction taking place in public in your community?
    - What do you think the benefits might be, of having someone reach out to you in the community to inform you that may have been exposed to TB and screened you for signs and symptoms of TB?
        - What else?
- If <u>you</u> had been exposed to someone with TB at a community venue/location by someone you didn't know, would it have been acceptable for someone to reach out to you in the community (for example at a shop, at a bar, or at a minibus stand), screen you for TB, and provide you TB-related education?
    - What makes you say that?
- What sorts of things would make [have made] this strategy more acceptable to you?
    - What else?
- Would the type of person who reached out to you the community and screened you for TB signs and symptoms matter to you?
    - For example… a trained community member that was a TB survivor vs. a health care worker?
    - What if instead they were a community leader or venue owner, such as a minibus driver, bar owner or pastor?
    - The gender of the person?
    - The age of the person?
- Would the location or venue where a person who reached out to you in the community and screened you for TB signs and symptoms matter to you?
    - Other than your home, are there certain places in the community that would be more acceptable for someone to approach you and screen you for TB symptoms?
- Would the time of day or day of the week for a person to reach out to you in the community and screen you for TB signs and symptoms matter to you?
    - Can you tell me more?
- Rather than have someone reach out to you directly, what if community venues had small paper slips - such as this one [show participant the contact invitation and allow them to

examine it] - available that said: 1) you may have been exposed to someone with TB and 2) here is where to go and what to do if you have any of these symptoms?
    - Can you tell me more?
- Of the 2 strategies I mentioned, can you please rank them in order of most preferred and least preferred? (e.g., (a) someone approaches you at a community venue/location, screens you for TB symptoms and answers any questions or, (b) you receive a piece of paper at a community venue that provides TB information and where to go if you have symptoms?
    - Can you walk me through your reasons for those preferences? [Why did you rank x option highest? Why did you rank x option lowest?]
    - Which of these would you definitely participate in?
    - Which would you definitely not participate in?
- After you began experiencing symptoms of your TB illness, what sorts of things made it difficult or challenging for you to quickly attend a health facility for further evaluation?
    - What else?
- After you began experiencing symptoms of your TB illness, what sorts of things might have made it easier to quickly attend a health facility for further evaluation?
    - What else?
    - How would you feel [have felt] about a small amount of money to cover the cost of transport to the facility?
    - How would you feel [have felt] if you did not have to wait in the queue at the facility and could immediately tested for TB without waiting (e.g., fast track)?
- What if instead of visiting a health facility to get tested for TB, you could have instead provided a sample for TB testing (for example sputum or urine) at a convenient community location with short wait times? Would this have been preferable?
    - Can you tell me more?
- What concerns would you have had about sharing information with healthcare workers about your close contacts and the places in the community you had recently visited?
    - What else?
    - Do your concerns about sharing your close contacts differ from your concerns about sharing the places you frequently visited in the community prior to your TB diagnosis?
    - What sorts of things would make [have made] it easier for you to share this type of information?
- In order to improve TB diagnosis in this community, we are considering a program in which recently diagnosed TB patients like yourself are asked to reach out to their close colleagues, friends and family members to encourage them to get screened for TB and provide them further information about where to get tested.
    - What kinds of thoughts and feelings would have about such a program?
    - How would you specifically feel if you were asked to contact your friends, colleagues and loved ones about their risk for TB and encourage them to be evaluated for TB at a health facility? [tell me more]

  - What sorts of things would make it easier for you to speak with your friends and colleagues about being at risk for TB and encouraging them to get evaluated for TB? [tell me more]
  - How would you have felt if you were asked to pass out a small paper card such as this one to your close contacts [show participant the contact invitation and allow them to examine it]? It provides information on TB symptoms and where to go for further evaluation. [tell me more]
  - What if you were provided a small amount of money for each close contact (friend, colleague or family member) you referred? [tell me more]
- In order to improve TB diagnosis in this community, we are also considering a program in which recently diagnosed TB patients <u>like yourself</u> are trained to become TB ambassadors. They would receive training and be provided a salary in order to reach out to community members at locations in George/Matero where TB transmission is most likely occurring. They would screen community members at these locations for TB symptoms and help provide them information about TB and where to go for help if they are ill. What are your thoughts about such a program?
  - Can you tell me more?
  - What factors would make you interested in participating in this program?
  - What additional information would you want to know before deciding whether or not to participate?
  - What concerns might you have about participating in such a program?
- What recommendations or suggestions do you have for making it easier to reach individuals in George/Matero to inform them that may have been exposed to TB disease and to then see if they may have any signs of symptoms of TB?
  - What else?

Wrap-up

- We have talked about many things related to TB. Are there any final thoughts you would like to share with me?
- Do you have any questions?

Thank participant for their time and contribution to the study.

Peer-to-peer TB study (P2PTB)

At-risk community members In-Depth Interview (IDI) Guide

Introduction

- How long have you lived in George/Matero?
- Tell me about your daily activities?
    - What community venues and places do you typically go to?
    - What types of places in the community might you visit at night or on the weekend? [tell me more]
- How often do you visit this venue/location in a typical week?
    - When you visit, how much time do you usually spend here?
- What are the top 2 health needs of persons in this community?
    - If TB mentioned: what makes you say TB?
    - If TB not mentioned: In your opinion, is TB an important issue in this community? [tell me more?]

TB Knowledge Attitude and Beliefs

- What do you know about TB?
    - What had you heard?
    - From whom?
    - What else have you heard?
- In your opinion, should awareness about TB be raised in George/Matero?
    - What makes you say that?
- Have any of your friends, colleagues or loved ones ever had TB?
    - Who?
    - How recently?
    - Anyone else?
    - How has this influenced what you think about TB?
- How do you think most people in this community get TB?
    - If places/venues <u>not</u> mentioned: <u>Where</u> do you think most people in this community get TB?
    - Anywhere else (outside of their home)?

TB Risk Perception

- How worried are you that you could get TB?
    - What makes you say that?
    - What sorts of things might make you think that you had TB?
- What would you do if you thought you had TB?
    - Where might you go?

Preferences for and concerns related to non-household contact tracing

Prompt/transition: I'd like to now ask you about some potential strategies for improving TB diagnosis in George/Matero.

- Imagine that you were in contact with a colleague, friend or family member who had recently been diagnosed with TB. What sorts of thoughts and feelings would you have?
    - Can you tell me more about that?
- Now, imagine that that person (colleague, friend or family member) directly reached out to you to inform you that they had been diagnosed with TB and recommended that you get tested for TB if you had any signs or symptoms of TB.
    - How would you feel about being contacted by that person directly?
        i. What makes you say that?
    - What would be your biggest concern about being contacted by that person directly?
        i. What else?
    - What do you think the benefits might be, of having a colleague, friend or family member reach out to you directly to inform of their TB diagnosis and recommend you get tested for TB if you have any signs or symptoms of TB?
        i. What else?
- How acceptable would such a strategy be to you?
    - What makes you say that?
- What sorts of things would make this strategy more acceptable to you?
    - What else?
- Would it make a difference whether your colleague, friend or family member did this face-to-face, or via phone/SMS?
    - What makes you say that?
- Next, imagine that you were exposed to TB somewhere outside of your home, by someone you did not know (for example, at a bar, shop, or in a minibus). Now imagine, if someone you didn't know approached you somewhere in the community (for example at a shop, at a bar, or at a minibus stand), to inform that you had recently been in contact with someone with TB and screened you for signs and symptoms of TB.
    - What would be your biggest concern about that?
        i. What else?
    - What specific concerns would you have about this interaction taking place in public in your community?
    - What do you think the benefits might be, of having someone reach out to you in the community to inform you that may have been exposed to TB and screened you for signs and symptoms of TB?
        i. What else?
- How acceptable would such a strategy be to you?
    - What makes you say that?
- What sorts of things would make [have made] this strategy more acceptable to you?
    - What else?
- Would the type of person who reached out to you the community and screened you for TB signs and symptoms matter to you?

    - For example… a trained community member that was a TB survivor vs. a health care worker?
    - What if instead they were a community leader or venue owner, such as a minibus driver, bar owner or pastor?
    - The gender of the person?
    - The age of the person?
- Would the location or venue where a person who reached out to you in the community and screened you for TB signs and symptoms matter to you?
    - Other than your home, are there certain places in the community that would be more acceptable for someone to approach you and screen you for TB symptoms?
- Would the time of day or day of the week for a person to reach out to you in the community and screen you for TB signs and symptoms matter to you?
    - Can you tell me more?
- Rather than have someone reach out to you directly, what if community venues had small paper slips - such as this one [show participant the contact invitation and allow them to examine it] - available that said: 1) you may have been exposed to someone with TB and 2) here is where to go and what to do if you have any of these symptoms?
    - Can you tell me more?
- Of the 2 strategies I mentioned, can you please rank them in order of most preferred and least preferred? (e.g., (a) someone approaches you at a community venue/location, screens you for TB symptoms and answers any questions or, (b) you receive a piece of paper at a community venue that provides TB information and where to go if you have symptoms?
    - Can you walk me through your reasons for those preferences? [Why did you rank x option highest? Why did you rank x option lowest?]
    - Which of these would you definitely participate in?
    - Which would you definitely not participate in?
- If you had symptoms that could be TB and testing at a health facility was recommended, what sorts of things might make it difficult or challenging for you to quickly attend a health facility for further evaluation?
    - What else?
- If you had symptoms that could be TB and testing at a health facility was recommended, what sorts of things might make it easier to quickly attend a health facility for further evaluation?
    - What else?
    - How would you feel about receiving a small amount of money to cover the cost of transport to the facility?
    - How would you feel if you did not have to wait in the queue at the facility and could be immediately tested for TB without waiting?
- What if instead of visiting a health facility to get tested for TB, you could instead provide a sample for TB testing (for example sputum or urine) to a trained peer or healthcare worker at a convenient community location with short wait times and you were then contacted by phone or SMS with the results and further instructions. What kinds of thoughts or feelings would you have about that?

    - What else?
- What recommendations or suggestions do you have for making it easier to reach individuals in George/Matero to inform them that may have been exposed to TB disease and to then see if they may have any signs of symptoms of TB?
    - What else?

Wrap-up

- We have talked about many things related to TB. Are there any final thoughts you would like to share with me?
- Do you have any questions?

Thank participant for their time and contribution to the study.

Peer-to-peer TB study (P2PTB)
Community venue owners and leaders In-Depth Interview (IDI) Guide

Introduction

- Can you please tell me about your job and what your role there is?
    - [where appropriate] Who do you report to?
- If owner: How long have you been running this shop/facility/venue?
- If not owner: How long have you been working at this shop/facility/venue?

Community venue social dynamics

- Can you tell me a little bit about what sorts of people tend to visit your shop/facility/venue?
    - Would you say is it mostly men or women?
    - What age groups?
    - Anything else that stands out about the sorts of people who are your customers?
- Who would you consider a regular customer to this shop/facility/venue?
    - How often must they visit? Any particular time?
    - Tell me more?
- When do you find your shop/facility/venue most busy?
    - Times of the day and days of the week?
    - What about the least busy?
- In your estimation, how many people visit your shop/facility/venue each day?
    - How many visitors might there be on a busy day?
    - How many visitors might there be on a non-busy day?
- In your estimation, what is the largest number of persons that might be in your shop/facility/venue at a given time on your busiest day?
- On a usual day, about how much time might a person spend at your shop/facility/venue?
    - What is the longest amount of time someone might spend visiting your shop/facility/venue?

TB knowledge, attitudes and beliefs

- What are the top 2 health needs of persons in this community?
    - If TB mentioned: what makes you say TB?
    - If TB not mentioned: In your opinion, how important is the issue of TB in this community? [Tell me more about that]
- What do you think about TB?
- What do you think about people who have TB?
- What things have influenced how you think about TB?
    - Knowing anyone who has had TB? Such as family friends, neighbors or coworkers?
    - HCW, interpersonal conversations, mass media, etc
- How do you think most people in this community get TB?
    - If places/venues <u>not</u> mentioned: <u>Where</u> do you think most people in this community get TB?

    - Anywhere else (outside of their home)?
    - Does this differ between men and women?
    - What makes you say that?
- In your opinion, should awareness about TB be raised in George/Matero?
    - What makes you say that?
    - Would you be interested in receiving education and training related to TB?
- How much do you worry that you could get TB?
    - Can you tell me more about that?
- How much do you worry that individuals attending your venue/facility could have undiagnosed TB and expose either yourself or other attendees?
    - Can you tell me more about that?
- In your opinion, in George/Matero, should new or better ways be identified to find more persons with TB and find them sooner
    - What makes you say that?

Preferences for and concerns related to non-household contact tracing

Prompt/transition: I'd like to now ask you about some potential strategies for improving TB diagnosis in George/Matero.

One strategy we are exploring is for healthcare workers to ask newly diagnosed TB patients where in the community they spend most of their time. Then we would visit those locations to screen individuals for signs and symptoms of TB.

- Imagine if someone with TB said that they had spent a large amount of time at your venue/facility/shop before being diagnosed. What sorts of thoughts and feelings would you have?
    - Can you tell me more about that?
- Now, imagine if someone came to <u>your</u> facility on a few different occasions to screen your patrons/attendees for signs and symptoms of TB. What would be your biggest concern about that?
    - What else?
- What do you think the benefits might be, of having someone come to your facility on a few different occasions to screen your patrons/attendees for signs and symptoms of TB?
    - What else?
- Under what conditions would you allow TB screening to be undertaken at your venue/facility/shop?
    - What makes you say that?
- What sorts of things would this strategy more acceptable to you?
    - What else?
    - What if a small cash incentive were to be offered for participating? Do you think that would make you more likely to participate, or would it not make any difference to you?
- Would the choice of time of day or day of the week to screen patrons/attendees at your venue/facility/shop matter to you?
    - Can you tell me more?

- Would the choice of person who came to your venue/facility/shop to screen patrons/attendees matter to you?
    - For example… A trained community member that was a TB survivor vs. health care worker?
    - The gender of the person?
    - The age of the person?
- If someone came to your facility to screen your patrons/attendees for signs and symptoms of TB, what things might make your patrons/attendees concerned about participating?
    - What else?
    - What things might make them interested in participating?
    - What else?
- Now, imagine if community members (for example, TB survivors) were trained to undertake TB screening at public community venues in George/Matero such as your venue/facility/shop. In your opinion, how effective would that approach be?
    - Can you please tell me more about that?
    - What would the benefits be of that approach?
    - What might be the drawbacks?
- Rather than have someone come to your venue/facility/shop to screen individuals for TB symptoms, how would you feel if we asked you to hand out small paper slips - such as this one [show participant the contact invitation and allow them to examine it] - to attendees such as this one that said: 1) you may have been exposed to someone with TB and 2) here is where to go and what to do if you have any of these symptoms?
    - Can you tell me more about that?
    - What would the benefits be of that approach?
    - What might be the drawbacks?
- Rather than have someone come to your venue/facility/shop to screen individuals for TB symptoms, how would you feel if you or your employees were provided brief education and training related to TB and worked with us to screen your patrons/attendees for signs and symptoms of TB?
    - In your opinion, how effective would that approach be?
    - How might it affect your business, either negatively or positively?
- Of the 3 strategies for screening patrons/attendees of your venue/facility/shop for signs and symptoms of TB can you please rank them in order of most preferred to least preferred? (e.g., (a) someone such as a trained community member who was a TB survivor comes to your facility and screens them, (b) you hand out a piece of paper that provides TB information and where to go if symptomatic, (c) you and/or your employees are provided a brief training and you screen patrons/attendees yourself?
    - Can you walk me through your reasons for that rank order? [Why did you rank x option highest? Why did you rank x option lowest?]
    - Which of these would you definitely participate in?
    - Which would you definitely not participate in?
- What additional recommendations or suggestions do you have for making it easier to reach individuals in George/Matero to inform them that they may have been exposed to TB disease and to then see if they may have any signs or symptoms of TB?

Wrap-up

- We have talked about many things related to TB. Are there any final thoughts you would like to share with me?
- Do you have any questions?

Thank participant for their time and contribution to the study.

Peer-to-peer TB study (P2PTB)
TB Providers In-Depth Interview (IDI) Guide

Introduction

- Can you tell me about your job and what your role at this facility is?

TB Knowledge and Attitudes

- How do you think most people in Geoge/Matero get TB?
    - At what places and businesses in George/Matero (other than in their house) do you think most people get exposed to TB?
    - What makes you say that?
    - Where else?
- In your opinion, should awareness about the TB be raised in George/Matero?
    - What makes you say that?
- In your opinion, in George/Matero, should new or better ways be identified to find more persons with TB and find them sooner?
    - What makes you say that?

Recommendations for and concerns related to non-household contact tracing

Transition: As mentioned before, we are very interested in understanding how to design a program to identify more people in the community and identify them sooner.

- Are there any programs or interventions beyond household contact tracing that you think could improve TB detection and should be implemented?
    - Can you describe that intervention?
    - What do you like about this intervention/strategy?

One strategy we are considering is to design a program to undertake TB screening among non-household contacts, including at community venues where TB transmission is suspected to occur.

- What advantages might this program have compared to existing services?
    - Tell me more?
- What disadvantages might this program have compared to existing services?
    - [if applicable] How might this compare to the strategy to improve TB detection that you suggested above?
- What reasons might make a new TB patient willing to share their close contacts outside of the home or places they had recently visited in the community?
    - Anything else?
- What reasons might make a new TB patient not willing to share their close contacts outside of the home or places they had recently visited in the community?
    - Anything else?
    - How may the reasons for not sharing close contacts differ from not sharing the venues they have visited?
        - Why might this be?

- If a TB patient was concerned about sharing information related to their close contacts and/or community venues visited, how might you encourage them to share this information?
  - Anything else?
  - What specific language might you use?
- If you were asked to design a program to find and screen community members for TB at different community venues – such as bars, shops, churches or minibus stands - how would you go about it?
  - How complicated or challenging do you think this would be?
  - Can you tell me more?
- If a trained TB peer (like the ones used for household contact tracing) approached community members at a public place/venue (for example a bar, shop or church) to inform them that they may have been exposed to TB and then asked to screen them for TB symptoms, what sorts of thoughts and feelings do you think community members may have?
  - What barriers might community members face to participating in TB screening at public community venues?
  - What strategies might we use to protect their privacy/confidentiality?
- What sorts of thoughts and feelings do you think owners and leaders of community venues would have if we undertook TB screening among attendees at their venue/business?
  - How might this differ by community venue type (e.g., bars, shop, minibus stand, church)?
  - How might we make this strategy more acceptable to them?
- If we were going to undertake TB contact tracing among non-household contacts at community venues/places in George/Matero, who are the key influential individuals to get on board with this strategy?
  - What makes you say that?
  - Anyone else?
- In your opinion, do you think using trained peers from the local community to undertake TB screening at public community venues in George/Matero could be effective?
  - What makes you say that?

Transition: In other countries, newly diagnosed TB patients have been asked to reach out directly to their close contacts such as friends, colleagues and family members to provide them with a paper slip like this one [show participant the contact invitation and allow them to examine it] – it says: 1) that they may have been in contact with someone with TB and 2) tells them where to go and what to do if they have any TB symptoms.

- What types of thoughts and feelings do you think new TB patients would have about this strategy?
  - What advantages might this strategy have compared to existing services?
  - What disadvantages might this strategy have compared to existing services?
  - What barriers might TB patients face to participating in such a strategy?
- Rather than having trained peers undertake TB screening at community venues, what if instead, venues where TB transmission is suspected to occur handed out paper slips similar to this one [show participant the contact invitation and allow them to examine it] in

order to inform community members: 1) they may have been exposed to TB and 2) here is where to go and what to do if they have any symptoms?
  - What advantages might this have compared to using trained peers to undertake TB screening at community venues?
  - What disadvantages might this have compared to using trained peers to undertake TB screening at community venues?
- Based on your experience and expertise, what additional recommendations or suggestions do you have for making it easier to reach individuals in George/Matero to inform them that may have been exposed to TB and to then see if they may have any signs of symptoms of TB?
  - Anything else?

Wrap-up

- We have talked about many things related to TB. Are there any final thoughts you would like to share with me?
- Do you have any questions?

Thank participant for their time and contribution to the study.

Peer-to-peer TB study (P2PTB)
TB Peers In-Depth Interview (IDI) Guide

Introduction

- How long have you worked as TB peer and helped with household contact tracing?
- What factors made you want to become a TB peer?
    - Before deciding to become a TB peer, what concerns did you have about the position?
- Have you previously had TB disease yourself?
    - If yes – how did this influence your decision to become a TB peer?
    - If yes – how has this affected your ability to communicate with persons who have exposed to TB and may be at risk for TB?

Understanding processes and challenges associated with household contact tracing

- Can you walk me through what a typical day is like for you?
    - What are your job responsibilities?
    - What meetings do you usually attend?
    - If yes - who else typically attends?
- What aspects of tracing and screening household contacts currently works well?
    - What else?
    - Are there specific approaches you've had good success with?
- What aspects of tracing and screening household contacts do you find to be the most challenging?
    - What else?
    - What changes have you made over time that has made aspects of your job easier or more successful?
    - Anything else?
- What do you do when you face challenges in your job?
    - Who do you talk with when you have challenges or difficulties with your job?
- How do you ask newly diagnosed patients about their recent contacts (e.g., what language do you use)?
    - What do you do when TB patients don't want to share their contact information?
    - Are there specific approaches you've had good success with in such cases?
    - What specific language might you use to encourage TB patients to share this information with you?
- What do you do when their household contacts don't want to participate with TB symptom screening?
    - Are there specific approaches you've had good success with?
    - What specific language might you use to encourage household contacts to participate in TB screening?
- What do you do when symptomatic contacts don't want to present to facilities for further evaluation and testing for TB?
    - Are there specific approaches you've had good success with?

    - What specific language might you use to encourage household contacts with TB symptoms to get tested?
- What sorts of things would make it easier to do your job and be more successful?
    - What else?
- If you were in charge for the day, what changes would you make to how household contacts are traced and screened for TB?
    - What makes you say that?
    - What else?

TB Knowledge and Attitudes

- How do you think most people get TB?
    - At what places and businesses in George/Matero (other than in their house) do you think most people get exposed to TB?
    - What makes you say that?
    - Where else?
- In your opinion, should awareness about the TB be raised in George/Matero?
    - What makes you say that?
- In your opinion, in George/Matero, should new or better ways be identified to find more persons with TB and find them sooner?
    - What makes you say that?

Recommendations for and concerns related to non-household contact tracing

Transition: As mentioned before, we are very interested in understanding how to design a program to identify more people in the community and identify them sooner.

- Are there any programs or interventions beyond household contact tracing that you think could improve TB detection and should be implemented?
    - Can you describe that intervention?
    - What do you like about this intervention/strategy?

One strategy we are considering is to design a program to undertake TB screening among non-household contacts, including at community venues where TB transmission is suspected to occur.

- What advantages might this program have compared to existing services?
    - Tell me more?
- What disadvantages might this program have compared to existing services?
    - [if applicable] How might this compare to the strategy to improve TB detection that you suggested above?
- What reasons might make a new TB patient willing to share their close contacts outside of the home or places they had recently visited in the community?
    - Anything else?
- What reasons might make a new TB patient not willing to share their close contacts outside of the home or places they had recently visited in the community?
    - Anything else?
    - How may the reasons for not sharing close contacts differ from not sharing the venues they have visited?

        - Why might this be?
- If you were asked to find and screen community members for TB at different community venues – such as bars, shops, churches or minibus stands - how would you go about it?
  - How complicated or challenging do you think this would be?
  - How confident are you that you could undertake TB screening at community venues and businesses?
  - What gives you that level of confidence (or lack of confidence)?
- If a trained TB peer like yourself approached community members at a public place/venue (for example a bar, shop, church or minibus stand) to inform them that they may have been exposed to TB and ask to screen them for TB symptoms, how do you think they would respond?
  - What barriers might community members face to participating in TB screening at public community venues?
  - What strategies might we use to protect their privacy/confidentiality?
- What sorts of thoughts and feelings do you think owners and leaders of community venues would have if you undertook systematic TB screening among attendees at their venue/business?
  - How might we make this strategy more acceptable to them?
  - Anything else?
- If we were going to undertake TB tracing among non-household contacts at community venues/places in George/Matero, who are the key influential individuals to get on board with this strategy?
  - What makes you say that?
  - Anyone else?
- In your opinion, do you think using trained peers (like yourself) to undertake TB screening at public community venues in George/Matero could be effective?
  - What makes you say that?

Transition: In other countries, newly diagnosed TB patients have been asked to reach out directly to their close contacts such as friends, colleagues and family members to provide them with a paper slip like this one [show participant the contact invitation and allow them to examine it] – it says: 1) that they may have been in contact with someone with TB and 2) tells them where to go and what to do if they have any TB symptoms.

- What types of thoughts and feelings do you think new TB patients would have about this strategy?
  - What advantages might this have compared to using trained peers like yourself to undertake TB screening at community venues?
  - What disadvantages might this have compared to using trained peers like yourself to undertake TB screening at community venues?
  - What barriers might TB patients face to participating in such a strategy?
- Rather than having trained peers like yourself undertake TB screening at community venues, what if instead, venues where TB transmission is suspected to occur handed out paper slips similar to this one [show participant the contact invitation and allow them to examine it] in order to inform community members: 1) they may have been exposed to TB and 2) here is where to go and what to do if they have any symptoms?
  - What advantages might this have compared to using trained peers to undertake TB screening at community venues?

    - What disadvantages might this have compared to using trained peers to undertake TB screening at community venues?
- Based on your experience and expertise, what additional recommendations or suggestions do you have for making it easier to reach individuals in George/Matero to inform them that may have been exposed to TB disease and to screen them for any signs of symptoms of TB?
    - Anything else?

Wrap-up

- We have talked about many things related to TB. Are there any final thoughts you would like to share with me?
- Do you have any questions?

Thank participant for their time and contribution to the study.
